# Policy cooperation and interface issues

**DOI:** 10.1186/2052-3211-8-S1-E3

**Published:** 2015-10-05

**Authors:** Sabine Vogler, Nina Zimmermann, Veronika J Wirtz, Zaheer-Ud-Din Babar

**Affiliations:** 1 WHO Collaborating Centre for Pharmaceutical Pricing and Reimbursement Policies, Health Economics Department, Gesundheit Österreich GmbH (Austrian Public Health Institute), Vienna, 1010, Austria; 2 Department of Global Health, Boston University School of Public Health, Boston, MA 02118, USA; 3 School of Pharmacy, Faculty of Medical and Health Sciences, University of Auckland, Private Mail Bag 92019, Auckland, New Zealand

## 

To successfully meet new challenges, policy-makers may have to bridge between different stakeholders, countries, segments, products and types of health care. Issues regarding policy cooperation and interface management in this field may be related to bridging out-patient and in-patient sectors, medicines and medical devices (treatment packages), pharmaceutical pricing and reimbursement, international cooperation initiatives (e.g. joint procurement), international databases (e.g. price/utilization databases) and international comparisons and benchmarking (e.g. cross-country price/utilization comparisons).

## Interface management related to bridging out-patient and in-patient sectors

Currently, in many European countries, pharmaceutical policies are taken separately for the out-patient sector and the in-patient sector, which appear to be seen as two distinct systems by policy-makers. Differences in payment systems between the out-patient and in-patient setting is likely to incentivize payers to find arguments why medicinal treatment might be shifted to the other sector as they need to minimize the burden for the budget for which they are responsible. This may particularly be an issue for high-cost medicines [1].

The urgent need to improve this type of interface management has been identified in the European Commission's co-funded project 'Pharmaceutical Health Information System' (PHIS) running from 2008 until 2011 [2]. It has been followed up in the 2nd Pharmaceutical Pricing and Reimbursement Information (PPRI) Conference held in Vienna on 29 and 30 September 2011, with a special strand devoted to it. At a fringe meeting alongside the PPRI Conference, several Conference delegates expressed their interest in continuing working to improve medicine management at the interface of the out-patient and in-patient sectors. As a follow-up activity the seminar 'Interface Management of Pharmacotherapy' was organised in Stockholm in September 2012 to raise awareness about the issue and to share good practice examples [3].

However, there are few best practice examples to improve interface management that is also known under different terms, such as seamless care, integrated care, transmural care and continuity of care [4]. The different terms are highlighting the novelty and limited evidence base in this field. Measures set at a micro-level of individual hospitals consist of cooperation with out-patient carers, including interventions at admission and particularly hospital discharge [5]. At the system level where the organisation and funding of the pharmaceutical system could be addressed, measures would imply legal and organisational changes such as joint reimbursement lists for both out-patient and in-patient sectors and joint Drugs and Therapeutics Committees with representatives from both sectors. However, only a few European countries have such policies in place [2,6]. In the Stockholm healthcare region in Sweden, a list of essential medicines recommendations (called the 'Wise List') valid for the out-patient and in-patient sectors is annually decided by a Joint Drugs and Therapeutics Committee (DTC) [7]. In Scotland, joint lists of recommended medicines for primary and hospital care have been present for over 20 years, with an involvement of both primary and secondary care physicians in the DTCs and in developing joint guidance and guidelines [8]. At the 2015 PPRI Conference Ken Paterson (University of Glasgow, K6) gives a key note in strand 3 and presents the experience in Scotland with joint formulary committees.

## Treatment packages of medicines and medical devices

Medical devices are playing an increasing role in medical treatment, in particular within the concept of 'personalised medicine' (or 'targeted medicine') which has gained increasing interest and funding including by the European Union.

However, in most countries, medical devices tend to be much less strictly regulated than medicines. Free pricing is usually applied to medical devices. Since there are no reimbursement mechanisms for medical devices, costs are, in principle, borne by patients or in the case of hospital care by hospitals. When it comes to the 'treatment package' composed of a medicine for treatment and a medical device for diagnostic purposes, substantial differences have been identified between European countries that have reimbursement systems for combined diagnostics and therapeutics (e.g. Germany, the UK and France), whereas for other countries (e.g. the Netherlands, Finland and Norway) no clear pathways for evaluation and funding of personalized medicine were identified [9]. An example can be found, for instance, in the treatment of breast cancer, with trastuzumab and diagnostics [10].

## Collaborative approaches

Recent developments have, once again, highlighted the need for further cooperation between countries and stakeholders. Over the last years, the cooperation between competent authorities of European countries has been strengthened, thanks to EU initiatives such as the High Level Pharmaceutical Forum (2006-2008) [11] and the Platform on Access to Medicines (2010-2013) [12] and voluntary initiatives, such as the PPRI project ([13], see also E4). A major challenge for Member States cooperation was sofosbuvir for the treatment of hepatitis C, with its high price challenging the financial sustainability of publicly funded health care systems. In his key note at the 2015 PPRI conference (strand 3), Florent Dromzée (French Ministry of Health) draws lessons from the envisaged cooperation between EU Member States in the 'sofosbuvir case'.

In this context, supportive approaches through scientific evidence and cooperation between authorities and payers are highly valuable, and should go beyond the issue of pricing and reimbursement alone, but also consider pre-launch and post-launch activities (see [1], K2, P8). At the PPRI conference, Anna Nachtnebel (Ludwig Bolzmann Institute for HTA, Austria, O15) demonstrates the value of collaborative models for increasing efficiency of early assessment of medicines, at the example of oncology medicines, and Wim Goetsch (National Health Care Institute, the Netherlands, O13) highlights the need for strengthening cooperation between HTA agencies, taking sofosbuvir as an example.

The 2015 PPRI conference coincides with the 30-year anniversary of the Nairobi Conference on the Rational Use of Drugs (see also K3). The Essential Medicines List (EML) is an important tool to prioritise medicines reimbursement, as part of Universal Health Coverage programs in countries; however, the criteria for inclusion into EML have been an issue of discussion given the recent inclusion of highly effective and high-priced medicines in EML (O14).
